# Understanding the factors behind non-adherence to pesticide safety guidelines among smallholder farmers in Fogera and Mecha districts, northwestern Ethiopia

**DOI:** 10.1186/s13104-025-07217-z

**Published:** 2025-04-16

**Authors:** Geteneh Mitku Chekol

**Affiliations:** Ethiopian Institute of Agricultural Research, Fogera Center Address, P.O. BOX 1937, Bihar Dar, Ethiopia

**Keywords:** Pesticide handling practices, Farmer barriers, Health and environmental safety, Agricultural sustainability, Northwestern Ethiopia

## Abstract

**Objective:**

The objective of the research was to identify key factors influencing smallholder farmers’ why not follow the recommended pesticide safety practices in northwestern Ethiopia. The study conducted in 2020/2021 investigated factors influencing smallholder farmers’ adherence to recommended pesticide safety practices in northwestern Ethiopia. The survey involving 50 farmers in Fogera and 53 in Mecha assessed pesticide use practices and perceptions, aiming to reveal common challenges in pesticide management. Survey data highlighted significant pesticide application and barriers related to adopting personal protective equipment (PPE), proper pesticide storage, integrated pest management (IPM), and safe disposal of containers.

**Result:**

Statistical analyses indicated non-significant differences between districts regarding these practices, underscoring universal issues such as economic constraints, limited resource access, and inadequate awareness. Crops like maize, faba bean, and cabbage showed high pesticide usage rates in both districts without statistically significant differences, emphasizing their cumulative impact on food safety. This finding underscores the urgent need for comprehensive interventions. Measures such as subsidized PPE, infrastructure development for safe pesticide storage, enhanced educational campaigns, and strengthened regulatory frameworks are crucial to promote sustainable agricultural practices and mitigate health and environmental risks. In conclusion, the study identifies widespread barriers to effective pesticide management among smallholder farmers in Fogera and Mecha, including economic limitations and resource constraints. Addressing these challenges necessitates a multifaceted strategy that includes subsidized PPE, improved storage infrastructure, and intensified educational initiatives to foster sustainable practices and safeguard both human health and the environment.

**Supplementary Information:**

The online version contains supplementary material available at 10.1186/s13104-025-07217-z.

## Introduction

Pesticides are essential in modern agriculture, significantly enhancing crop productivity and ensuring global food security [1–3]. These chemicals are critical inputs in contemporary farming due to their high efficacy and reliability in protecting crops from pests, which consequently guarantees high crop yields [4–6].

However, their indiscriminate use poses significant risks to both the environment and human health, leading to acute and chronic health issues [7]. Researchers have documented that the annual incidence rates of acute pesticide poisoning can reach as high as 18 per 100,000 full-time agricultural workers and 7 per million among schoolchildren [8]. Additionally, chronic effects such as cancer have been observed in agricultural workers [9]. Pesticide exposure to humans and the environment can occur during mixing, loading, or application, as well as through contact with treated crops during field re-entry [10, 11].

Common unsafe practices in developing countries include overspray [12], lack of personal protective equipment [13], improper storage of pesticides and their containers [14], and the reuse of washed pesticide containers for food and drinking water. For instance, approximately 35% and 77% of farmers in Nigeria and Ethiopia, respectively, engage in these unsafe practices [15].

Farmers’ behaviors in pesticide use are influenced by several factors, including their perceptions [16, 17], gender and age [4, 18], level of knowledge, and the influence of pesticide retailers [19, 20]. Proper safety measures during the application and disposal of pesticides are crucial to mitigate these risks and protect both the environment and human health [7].

The consequences of pesticide exposure often arise when handlers neglect to wear Personal Protective Equipment (PPE) and engage in unsafe pesticide handling practices. Therefore, the utilization of proper PPE, selecting the appropriate type of gear, and employing safe handling practices can mitigate the risks associated with pesticide exposure [21].

In Ethiopia, as in many developing countries, pesticide use is prevalent in agriculture, driven by the need to combat pests and diseases that threaten crop yields. Despite legislative efforts such as the Pesticide Registration and Control Proclamation No. 674/2010 and other regulatory frameworks, challenges persist in ensuring safe pesticide handling practices [22–24]. Studies indicate gaps in knowledge regarding pesticide risks, inadequate awareness of safe handling practices, and poor adherence to hygiene standards among agricultural workers [25, 26].

The efficient use and disposal of pesticides are critical for minimizing environmental contamination and health risks associated with their misuse. However, the realities on the ground in Ethiopia often diverge from these ideal practices, with reports of improper pesticide storage, incorrect disposal of pesticide containers, and insufficient use of personal protective equipment (PPE) during application [27, 28]. These practices not only jeopardize agricultural sustainability but also pose significant health hazards to farmers and surrounding communities.

Therefore the research aims with the research questions, what a key factors influencing smallholder farmers’ adherence to recommended pesticide safety practices in northwestern Ethiopia? By addressing these questions, this research aims to contribute to the development of evidence-based strategies that promote safer pesticide use and mitigate the environmental and health risks associated with pesticide handling in Ethiopia’s agricultural sector.

## Main text

### Material and method

#### Description of the study area

**Fig. 1:**
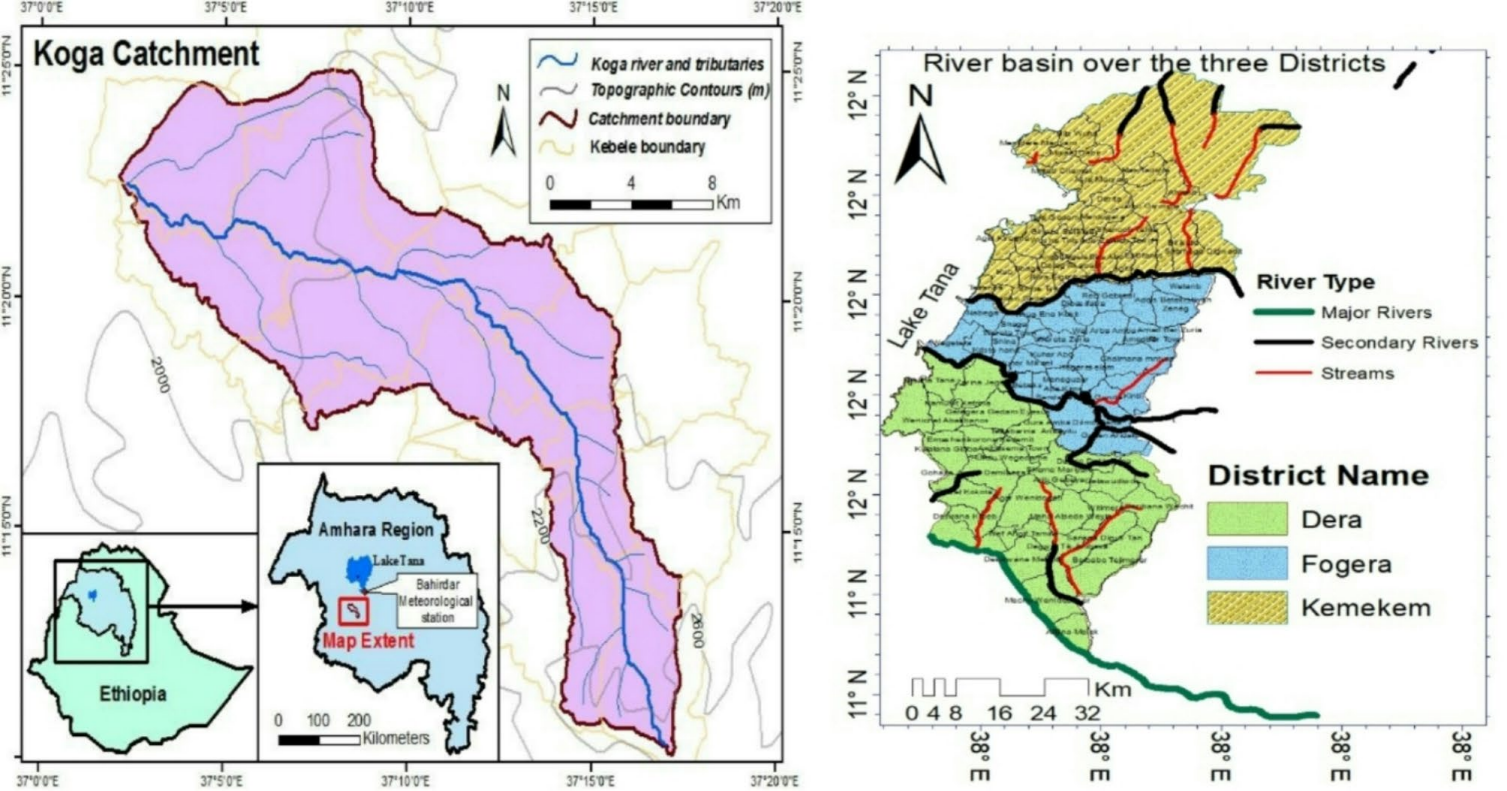
Map of Koga irrigation scheme and Fogera plain of vegetable growing areas covered by the survey

This research, which was carried out in 2020–2021, focusses on farmers’ poor pesticide use practice and associated factors. The experiment conducted particularly in the irrigation production season of Fogera Plain and Koga irrigation scheme of South Gondar Zone and North Gojam respectively, as described in Fig. 1. Koga is situated 35 km from Bahir Dar, close to Merawi town (11.35°N, 37.14°E, and 1900 m above sea level). The other study location located on the Fogera Plain which found the headwaters of the Blue Nile River, whose principal tributaries are the Gumara and Ribb Rivers, are. The coordinates of the Fogera district are 11.58°N and 37.41°E.

### Survey sampling procedure

The study’s data comes from a farmer perception survey conducted in the Mecha districts and Fogera Plain in 2020–2021. By observing inadequate pesticide management, we gathered information on usage, inappropriate storage, and empty container disposal. We asked 50 farmers in Mecha districts and 53 farmers in Fogera districts who used improper pesticide handling techniques why they did so. With possible survey sites provided by the district development office, the survey focused on household heads of irrigation vegetable farmers.

The Koga irrigation area and Fogera Plain were purposefully chosen for high pesticide usage for vegetable production through the use of a multi-stage sampling technique. Interviews were conducted with seven of the twelve irrigation blocks in Koga and seven of the kebeles in Fogera. Respondents were chosen at random via the transect technique outlined in [29]. Face-to-face interviews were held on the farms, making up the sample size.

### Survey questionnaire

There were 17 main questions on the form, and each interview lasted roughly 20 min. It contained multiple-choice and open-ended questions that were accurately constructed within the cultural context of farmers in order to understand the motivations behind illicit pesticide use practices.

### Collected data

Socioeconomic and lifestyle variables such as age, sex, education level, and land tenure were included in the data collection. Regarding health hazards associated with pesticide use in vegetable production, farmers were also questioned. The questionnaire addressed pest control and pesticide handling, emphasizing causes of incorrect behavior such as inadequate storage of pesticides, absence of safety gear, and inappropriate disposal of empty pesticide containers.

### Data analysis

Descriptive statistics (frequency distributions, percentages, means, and standard deviations) were calculated. The JASP software and Excel Microsoft were used to determine descriptive statistics and percentages.

## Result

### Socio demographic characteristics of respondents

Table 1 displays that the demographic characteristics of the respondents in Mecha and Fogera. 54% of responders in Fogera and 57% in Mecha are middle-aged. The level of education varies; respondents in Mecha (43%) were more illiterate than in Fogera (30%). Compared to Mecha (34%), Fogera had more responders (42%) with education levels in Grades 1–4. Mecha has 40% more small farms (less than 0.5 hectares) than Fogera (26%). Families in Mecha had a higher percentage of 7–11 members (51%) than those in Fogera (24%). Significant variations in family size are revealed by statistical testing (*p* = 0.015).

**Table 1:** Demographic characteristics of survey respondents (multiple responses possible)

Characteristic	Category	Farmers’ response *N* (%)		X²	*P* value
		Fogera (*N* = 50)	Mecha (*N* = 53)		
Age	Mid age	27 (54%)	30 (57%)	0.164	0.921
	Old	9 (18%)	8 (15%)		
	Young	14 (28%)	15 (28%)		
Educational level	Grade 9-college	3 (6%)	4 (8%)	2.446	0.485
	Illiterate	15 (30%)	23 (43%)		
	Grade 1–4	21 (42%)	18 (34%)		
	Grade 5–8	11 (22%)	8 (15%)		
Farm size	0.5-1 ha	13 (26%)	15 (28%)	3.14	0.209
	< 0.5 ha	13 (26%)	21 (40%)		
	> 1 ha	13 (26%)	17 (32%)		
Number of total family	1–3	10 (20%)	9 (17%)	8.4	0.015
	4–6	28 (56%)	17 (32%)		
	7–11	12 (24%)	27 (51%)		

Key = ns = not significant, * = significant at 0.05 level, ** = significant at 0.01 level

### Farmers pesticide use practice on different crops after harvest for grains and at maturity for vegetables

Before addressing the main questions about the reasons behind pesticide misuse, we first examined pesticide application practices on the primary crops grown in the study area. The results are summarized in Table 2 which provides insights into pesticide use across different crops. For finger millet, only 8% of respondents apply pesticides, indicating low pesticide use. Tef shows no pesticide use, which is consistent across different areas. Rice has 10% pesticide application, suggesting moderate use. Maize exhibits very high pesticide use, with 96% of respondents applying pesticides, including WHO classes I and II. Faba beans also have high pesticide use, with 66% of respondents applying pesticides. Chickpea shows substantial use, with 74% applying pesticides. Grass pea has high pesticide usage, with 88% of respondents applying pesticides. Onion and cabbage both show very high application rates, at 96% and 94%, and 91% respectively. These results reveal significant variation in pesticide application across different crops, highlighting some crops with notably high pesticide usage.

**Table 2:** Represents the farmers’ response for pesticide use on different crops after harvest for grains and at maturity for vegetables (*N* = 103)

Crop	District (Fogera, *N* = 50 and Mecaha, *N* = 53)	Do you apply pesticides for the grain (storage) and vegetables?		χ²	*p*-value	Pesticides used by the farmers	WHO toxicity class
		No (%)	Yes (%)				
Finger Millet	Fogera	46 (92%)	4 (8%)	4.41	0.04		
	Mecha	53 (100%)	0 (0%)				
Tef	Fogera	48 (96%)	0 (0%)	2.16	0.14		I, II
	Mecha	53 (100%)	0 (0%)				
Rice	Fogera	45 (90%)	5 (10%)	5.57	0.02		I, II
	Mecha						
Maize	Fogera	2 (4%)	48 (96%)	0.41	0.5	Methyl bromide, Malathion, Diazinon, DDT	I, II
	Mecha	1 (2%)	52 (98%)				
	Mecha	0	53 (100%)				
Faba bean	Fogera	17 (34%)	33 (66%)	0.39	0.5	Methyl bromide, Malathion, Diazinon,	I, II
	Mecha	15 (28%)	38 (72%)				
Chickpea	Fogera	13 (26%)	37 (74%)			Methyl bromide, Malathion, Diazinon	I, II
	Mecha						
Grass pea	Fogera	6 (12%)	44 (88%)	1.43	0.23	Methyl bromide, Malathion, Diazinon	I, II
	Mecha	11 (21%)	42 (79%)				
Onion	Fogera	2(4%)	48 (96%)	0.03	0.87	Dimethoate, Profenofos, Lambda-cyhalothrin, Deltamethrin	II
	Mecha	2 (4%)	48 (96%)				
Cabbage	Fogera	3 (6%)	47 (94%)	0.42	0.51	Dimethoate, Lambda-cyhalothrin, ProfenophoseMancozeb + Metalaxyl,	II, III
	Mecha	5 (9%)	48 (91%)				

Key ns = not significant, * = significant at 0.05 level, ** = significant at 0.01 level

### Barriers to PPE use in pesticide handling among smallholder farmers

According to Table 3, the main reasons why farmers are reluctant to use personal protection equipment (PPE) are because of its high cost, limited availability in markets, discomfort, and lack of understanding. 66% of farmers in Fogera and 71% in Mecha strongly believe that high costs are a barrier; there is no discernible difference between the two districts (*p* = 0.778). Regarding market access, there is no discernible difference (*p* = 0.71) between the percentage of farmers in Fogera and Mecha who strongly agree that PPE is unavailable (36% versus 28%). Concerns about discomfort are expressed by 46% of Fogera farmers and 36% of Mecha farmers, respectively, with no discernible difference (*p* = 0.33). Nonetheless, a noteworthy problem is ignorance, as indicated by the agreement of 70% of Fogera farmers and 57% of Mecha farmers, a difference that is only marginally significant (*p* = 0.09).

**Table 3:** Showed that why farmers do not use PPE (*N* = 103) (multiple responses possible)

Why farmers do not use PPE? (reason)	Districts (Fogera, *N* = 50 and Mecaha, *N* = 53)	Farmers’ response *N* (%)			Χ²	*p*-value
		Agree	Disagree	Strongly agree		
High cost	Fogera	14 (28%)	3 (6%)	33 (66%)	0.50	0.778^ns^
	Mecha	13 (25%)	2 (4%)	38 (71%)		
No access in the market	Fogera	16 (32%)	16 (32%)	18 (36%)	0.70	0.71 ^ns^
	Mecha	19 (36%)	19 (36%)	15 (28%)		
Uncomfortable to use	Fogera	23 (46%)	14 (28%)	13 (26%)	2.23	0.33 ^ns^
	Mecha	17 (32%)	17 (32%)	19 (36%)		
Lack of awareness	Fogera	35 (70%)	5 (10%)	10 (20%)	4.62	0.09*
	Mecha	30 (57%)	14(26%)	9(17%)		

Key: Significance Level (*): A 10% probability that results are due to random chance, used in hypothesis testing to assess statistical significance

### Barriers to proper pesticide storage among smallholder farmers

Table 4 illustrates how all responders improperly store pesticides by utilizing common household objects or improvised constructions. Regarding infrastructure (*p* = 0.480), separate storage (*p* = 0.232), health effects (*p* = 0.132), and security concerns (*p* = 0.630), there are no appreciable variations between the districts. A significant issue for 60% of farmers in Fogera and 53% in Mecha is inadequate storage. 54% of farmers in Fogera and 55% in Mecha are aware of the negative effects storage practices have on health. Security issues are noted even though the changes are not statistically significant (X2 values ranging from 0.925 to 4.044, all *p* > 0.05). These results point to recurring issues and point to the necessity of stronger infrastructure and instruction for safer pesticide storage.

**Table 4:** represents, why farmers do not store in the right place (*N* = 103) (multiple responses possible)

Why do you not store pesticide in the right place?	Districts (Fogera; *N* = 50) Mecha; *N* = 53)	Farmers’ response *N* (%)			Χ²	*p*-value
		Agree	Disagree	Strongly agree		
Limited infrastructure	Fogera	0 (0%)	28 (56%)	11 (22%)	2.475	0.48 ^ns^
	Mecha	1 (2%)	26 (49%)	17 (32%)		
Lack of other separate house	Fogera	30 (60%)	11 (22%)	9 (18%)	2.919	0.23 ^ns^
	Mecha	28 (53%)	8 (15%)	17 (32%)		
Ignore health effect	Fogera	27 (54%)	13 (26%)	10 (20%)	4.044	0.13 ^ns^
	Mecha	29 (55%)	20 (38%)	4 (8%)		
Security concerns	Fogera	22 (44%)	14 (28%)	14 (28%)	0.925	0.63 ^ns^
	Mecha	19 (36%)	19(36%)	15 (28%)		

Key: ns = not significant at *p* < 0.05 level

### Challenges in proper disposal of empty pesticide containers by farmers

Our study in Table 5 reveals that improper pesticide container disposal is common among farmers in Fogera and Mecha districts, with many washing sprayers and disposing of the discharge inappropriately. There is no significant difference between districts in factors like lack of awareness (*p* = 0.976), understanding of environmental impacts (*p* = 0.861), or enforcement of disposal laws (*p* = 0.991). Environmental risk concerns are significant but similar (Fogera: 28%, Mecha: 30%, Χ² = 1.18, *p* = 0.55). Awareness levels vary (Fogera: 28%, Mecha: 53%), but not significantly (Χ² = 0.79, *p* = 0.67). Uncertainty about impacts and concerns about enforcement are prevalent but not significantly different between districts (Fogera: 44%, Mecha: 49%, Χ² = 0.23, *p* = 0.861; Fogera: 54%, Mecha: 55%, Χ² = 0.02, *p* = 0.991). These findings highlight the need for improved awareness and enforcement to enhance disposal practices.

**Table 5:** Represents that the reason of farmers why not remove empty pesticide containers properly (*N* = 103)

Why do you not remove empty pesticide containers properly?	Districts (Fogera; *N* = 50) Mecha; *N* = 53)	Farmers’ response *N* (%)			Χ²	*p*-value
		Agree	Disagree	Strongly agree		
Ignore environmental risk	Fogera	14 (28%)	3 (6%)	33 (66%)	1.18	0.55 ^ns^
	Mecha	16 (30%)	1 (2%)	36 (68%)		
Lack of awareness	Fogera	14 (28%)	19 (38%)	17 (34%)	0.79	0.67 ^ns^
	Mecha	28 (53%)	18 (34%)	7 (13%)		
Do not know its impact	Fogera	22 (44%)	18 (36%)	10 (20%)	0.23	0.86 ^ns^
	Mecha	26 (49%)	18 (34%)	9 (17%)		
Absence of enforcing law	Fogera	27 (54%)	14 (28%)	9 (18%)	0.02	0.99 ^ns^
	Mecha	29 (55%)	15 (28%)	9 (17%)		

Key; ns (0.55): Not significant at *p* < 0.05 level

### The reasons behind why the small holder farmers do not follow right pesticide mixing practice

The findings in Table 6 show that a number of factors lead to farmers spraying pesticides at higher than advised amounts and combining chemical practices at home or near rivers. Although this justification is statistically non-significant (*p* = 0.674), a considerable percentage of farmers 50% and 45%, respectively agree or strongly agree that they combine techniques at home or near rivers because they disregard environmental hazards. Another explanation is a lack of appropriate direction, to which 45% of farmers overall agreed or strongly agreed. However, the results are not statistically significant (*p* = 0.203). 45% of farmers think that higher application rates of pesticides are more effective when compared to prescribed rates; a marginally significant p-value of 0.087 indicates that this opinion may be a major impact. Finally, 68% of farmers concur that they are unaware of the recommended rates; nonetheless, this explanation is not statistically significant (*p* = 0.813). These results demonstrate that, although disregarding environmental risks and lacking appropriate guidance do not substantially impact practices, the belief in the increased efficacy of pesticide rates above recommended levels exhibits a trend towards significance, suggesting the need for improved pesticide usage education.

**Table 6:** Represents the reasons why farmers do not follow the right practice of pesticide mixing (multiple responses are possible)

Pesticide mixing practice	Districts (Fogera; *N* = 50) Mecha; *N* = 53)	Farmers’ response *N* (%)			Χ²	*p*-value
		Agree (*N*= %)	Disagree (*N*= %)	Strongly agree (*N*= %)		
**Reasons for mixing pesticide in non-appropriate place**						
Miss Perceived Environmental Safety	Fogera	27 (54%)	18 (36%)	5 (10%)	0.79	0.674 ^ns^
	Mecha	24 (45%)	23 (43%)	6 (11%)		
Lack of Proper Guidance	Fogera	10 (20%)	35 (70%)	5 (10%)	3.19	0.203 ^ns^
	Mecha	13 (25%)	39 (74%)	1 (2%)		
**Reasons for Applying Above Recommended Pesticide Rates**						
Belief in Higher Effectiveness	Fogera	26 (52%)	14 (28%)	10 (20%)	4.884	0.087*
	Mecha	17 (32%)	17 (32%)	19 (36%)		
Unawareness of Recommended Rates	Fogera	19 (38%)	16 (32%)	15 (30%)	0.413	0.813 ^ns^
	Mecha					

Significance Key: ns: Not significant at *p* < 0.05 level and * represents the significant difference presence at *p* > 0.05 level

## Discussion

### Pesticide use practice in the study area

The research findings from multiple studies on pesticiadoption rates among farmers, in line with globalde residue levels in various crops support the assertion that crops like maize and faba beans are expected to have higher pesticide residues due to their high application rates [30]. Conversely, finger millet and tef are likely to have lower residue levels as they have very low pesticide use [31]. Rice falls in the middle with moderate pesticide use, potentially resulting in moderate residue levels [32]. Additionally, onion and cabbage, which also exhibit high application rates, may correlate with higher residue levels [30]. These insights emphasize the importance of understanding the pesticide application rates for different crops to predict and manage pesticide residue levels effectively, ensuring food safety and consumer health.

### Use of personal protective equipment (PPE)

The survey results indicate that various factors influencing farmers’ decisions not to use Personal Protective Equipment (PPE) do not significantly differ between districts, aligning with [2] on economic barriers as a major deterrent for smallholder farmers. The non-significant association between district and the high cost of PPE (p-value = 0.778) and the lack of access to PPE in local markets (p-value = 0.705) resonates with [23, 26], emphasizing challenges in accessing protective gear in rural areas. Additionally, discomfort (p-value = 0.328) and ignorance of health effects (p-value = 0.099) not significantly varying by district align with [8, 21] indicating universal concerns about practicality, comfort, and knowledge gaps regarding pesticide exposure risks among farmers. Educational initiatives and improved distribution channels for PPE are crucial for enhancing accessibility and adoption rates among farmers, in line with global regulations and directives from FAO, WHO, and EU [33–36].

### Pesticide storage practices

The survey also explored reasons for improper pesticide storage practices among farmers across different districts, revealing no significant association with the district for various factors including limited infrastructure, lack of separate storage houses, convenience, and economic constraints.

Firstly, the non-significant association between the district and limited infrastructure (p-value = 0.480) aligns with [26] highlighting widespread deficiencies in storage facilities among smallholder farmers. Similarly, the lack of significant association between district and absence of separate storage houses (p-value = 0.232) this finding implied that emphasizing the prevalent practice of storing pesticides in accessible locations due to the lack of dedicated storage facilities. Establishing designated storage areas is crucial to reducing risks associated with improper storage practices. The finding that convenience and economic constraints (p-value = 0.132 and 0.630, respectively) are not significantly associated with district aligns with studies and [15], indicating that practical considerations and financial limitations influence storage decisions. Interventions should focus on balancing practicality with safety to encourage proper storage practices. The results are consistent with previous findings, which highlighted that farmers lack knowledge about pesticide toxicity and safe handling. Additionally, there is no significant association between educational status and knowledge.

### Disposal of empty pesticide containers

The survey findings indicate that reasons for improper disposal of empty pesticide containers do not significantly differ between districts. Factors such as lack of awareness, disposal facilities, economic constraints, and cultural practices showed no significant association with district. The lack of significant association between district and lack of awareness (p-value = 0.976) aligns with previous research highlighting farmers’ insufficient knowledge about safe disposal practices [26]. Educational initiatives are crucial to promoting awareness and encouraging proper disposal behaviors. Similarly, the non-significant association between district and lack of disposal facilities (p-value = 0.861) reflects challenges identified by [15, 17], emphasizing the need for infrastructure development to support safe disposal practices.

The finding that economic constraints and cultural practices (p-value = 0.991) are not significantly associated with district aligns with studies by [3], indicating that economic factors and traditional habits influence farmers’ disposal practices universally.

### Pesticide mixing practices

Concerns about pesticide mixing near water sources and homes pose risks to health and the environment, affecting Lake Tana through contamination from local rivers [35–37]. In Fogera, 54% of respondents recognize these risks, while 45% in Mecha do. Awareness of proper mixing methods is limited, with 20% in Fogera and 25% in Mecha lacking knowledge. Regarding to the pesticide dosage 52% of Fogera respondents and 36% of Mecha respondents believe higher dosages are more effective. These findings suggest a need for targeted education to improve pesticide use practices [37].

## Conclusion

Farmers frequently lack the expertise necessary to handle pesticides safely, which can result in inappropriate container disposal, poor PPE use, and incorrect storage. Cultural norms, insufficient infrastructure, and financial limitations all play a part in these problems. Reducing health risks and ensuring safer food production require strengthening PPE access, encouraging IPM, offering suitable storage facilities, improving education on pesticide dangers, and making sure pesticide containers are disposed of properly. Through the use of consistent findings from prior research and diverse districts, agricultural practices can be improved and the dangers associated with pesticide use can be reduced through customized treatments.

### Strengthen and limitation

The small sample size of this study limits the findings’ generalizability and may not adequately reflect the variety of experiences found in Ethiopia’s many regions. In spite of this, the research has several noteworthy advantages, especially given its distinct focus. This research investigates the fundamental causes of pesticide usage and perceptions, in contrast to most studies that focus on current practices and myths. It offers useful insights for creating focused activities and regulations to enhance pesticide use and management going forward by identifying the causes and contributing elements of inappropriate behaviors. Further studies including a bigger, more varied sample size would support and build on these results.

## Electronic supplementary material

Below is the link to the electronic supplementary material.

Supplementary Material 1

## Data Availability

The datasets generated and/or analyzed during the current study are available from the corresponding author on reasonable request.
