# Supplementary material for: Understanding the factors behind non-adherence to pesticide safety guidelines among smallholder farmers in Fogera and Mecha districts, northwestern Ethiopia
Source: BMC Res Notes. 2025 Apr 16;18:177. doi: 10.1186/s13104-025-07217-z (PMC12004810; doi:10.1186/s13104-025-07217-z)
Supplement: Supplementary file 1 — Supplementary Material 1 [file 13104_2025_7217_MOESM1_ESM.docx]

**Understanding the Factors behind Non-Adherence to Pesticide Safety Guidelines among Smallholder Farmers in Fogera and Mecha Districts, Northwestern Ethiopia**

**Questionnaire**

**Section 1: Demographic Characteristics of Respondents**

1. **Age:**
   - Young (18-35 years)
   - Mid Age (36-55 years)
   - Old (56 years and above)
2. **Educational Level:**
   - Illiterate
   - Grade 1-4
   - Grade 5-8
   - Grade 9-College
3. **Farm Size:**
   - Less than 0.5 hectares
   - 0.5-1 hectare
   - More than 1 hectare
4. **Total Family Size:**
   - 1-3 members
   - 4-6 members
   - 7-11 members

**Section 2: Pesticide Personal Protective Equipment (PPE)**

1. **Why do you not use PPE when handling pesticides?** (Select all that apply)
   - High cost
   - No access in the local market
   - Uncomfortable to use
   - Lack of awareness/ignore health effects

**Response Options:**

- - Agree
  - Disagree
  - Strongly Agree

**Section 3: Pesticide Mixing Practices**

1. **Why do you apply inappropriate pesticide mixing practices?** (Select all that apply)
   - Mixing in a non-appropriate place
   - Lack of awareness about how to mix properly
   - Recommendations from others
   - Recent pesticides are not effective

**Response Options:**

- - Agree
  - Disagree
  - Strongly Agree

**Section 4: Pesticide Storage Practices**

1. **Why do you not store pesticides in the right place?** (Select all that apply)
   - Limited infrastructure for proper storage
   - Lack of a separate storage facility
   - Ignorance of health effects
   - Security concerns

**Response Options:**

- - Agree
  - Disagree
  - Strongly Agree

**Section 5: Child Labor in Pesticide Application**

1. **Why are children under 18 years spraying pesticides in your area?** (Select all that apply)
   - Labor shortages
   - Economic necessity
   - Lack of awareness/ignore health effects

**Response Options:**

- - Agree
  - Disagree
  - Strongly Agree

**Section 6: Integrated Pest Management (IPM)**

1. **Why do you not use Integrated Pest Management (IPM) for pest management?** (Select all that apply)
   - Lack of awareness about IPM
   - No IPM components available in the market
   - Consider IPM not practical
   - Not viewed as an effective alternative pest control method

**Response Options:**

- - Agree
  - Disagree
  - Strongly Agree

**Section 7: Disposal of Empty Pesticide Containers**

1. **Why do you not remove empty pesticide containers properly?** (Select all that apply)
   - Ignorance of environmental risks
   - Lack of awareness about proper disposal
   - Uncertainty about the impact of improper disposal
   - Absence of enforcing laws or regulations

**Response Options:**

- - Agree
  - Disagree
  - Strongly Agree

1. **Main Crop Grown:**

Finger Millet

Tef

Rice

Maize

Lentil

Faba Bean

Chickpea

Grass Pea

Onion

Tomato

Potato

Cabbage
